# Differential anaerobic oxidation of benzoate in *Geotalea daltonii* FRC-32

**DOI:** 10.1128/spectrum.02324-24

**Published:** 2025-03-05

**Authors:** Christina M. Kiessling, Sujay Greenlund, James E. Bullows, Cayden Samuels, Feranmi Aboderin, Nuria Ramirez, Kuk-Jeong Chin

**Affiliations:** 1 Department of Biology, Georgia State University, Atlanta, Georgia, USA; Ruhr-Universitat Bochum, Bochum, Germany

**Keywords:** *Geotalea daltonii*, benzoate, acetate, differential anaerobic degradation, carbon source availability, metabolic strategies

## Abstract

The efficient carbon source utilization in dynamic environments, including anoxic subsurface contaminated by aromatic compounds*,* is a challenge for anaerobic bacteria such as *Geotalea daltonii* strain FRC-32. The aim of this study was to elucidate the metabolic pathways employed by *G. daltonii* FRC-32 during anaerobic benzoate oxidation in the presence of acetate, a key intermediate in anaerobic organic matter degradation, to predict carbon source transport and utilization strategies. Simultaneous carbon source oxidation and monoauxic growth were observed in *G. daltonii* FRC-32 cultures grown on 1 mM benzoate + 5 mM acetate, 1 mM benzoate + 2 mM acetate, and 2 mM acetate spiked with 1 mM benzoate. Sequential carbon source oxidation and diauxic growth were observed only in cultures grown on 5 mM acetate spiked with 1 mM benzoate. Benzoate accumulation in *G. daltonii* FRC-32 whole cell lysates indicated that intracellular benzoate transport occurred during benzoate oxidation in the presence of acetate. Expression analyses of putative benzoate transporter BenK and protein-ligand binding affinity prediction suggested BenK’s specificity for transporting benzoate. Relative expression levels for the gene *benK,* encoding BenK, and the genes *bamNOPQ,* involved in the benzoyl-CoA pathway, were significantly higher in cultures grown on both benzoate and acetate than in cultures grown on acetate as sole carbon source, indicating that intracellular benzoate accumulation facilitated the regulation of *bamNOPQ*. Our results demonstrated that *G. daltonii* FRC-32 can perform differential benzoate oxidation in the presence of acetate, by either simultaneous or sequential carbon source oxidation, which indicated the metabolic plasticity of *G. daltonii* FRC-32 in response to varying carbon source availability.

**IMPORTANCE:**

The contamination of anaerobic subsurface environments by crude oil derivatives including aromatic compounds is a global concern due to the persistence and toxicity of these pollutants. Anaerobic bacteria play a crucial role in the degradation of aromatic hydrocarbons under anoxic conditions; however, the potential mechanisms involved in metabolic regulation of aromatic degradation pathways are not well understood. This study contributed to elucidating how *G. daltonii* strain FRC-32 efficiently utilizes benzoate as a carbon source in the presence of acetate. Findings of intracellular benzoate accumulation and regulation of key genes associated with benzoate oxidation contributed to the understanding of *G. daltonii* FRC-32’s aromatic degradation pathways, provided significant insights into potential mechanisms that modulate anaerobic benzoate oxidation in the presence of the energetically favorable carbon source acetate, and indicated metabolic strategies of *G. daltonii* FRC-32 in response to dynamic environmental conditions.

## INTRODUCTION

Remediating environments contaminated by aromatic compounds is an urgent issue, and bioremediation, the process of removing contaminants through microbial activity (1), has become more essential, especially in anaerobic subsurface environments (2–6). Benzoate has been widely used as a model compound for the study of bacterial catabolism of aromatic compounds (7, 8) as its degradation is initiated by activation to benzoyl-CoA, a key intermediate into which most aromatic compounds are metabolized via various channeling reactions (9–11). Our previous study (12) suggested that the benzoyl-CoA pathway plays a central role in *G. daltonii* strain FRC-32’s anaerobic metabolism of a plethora of aromatic compounds; upon initial activation, the peripheral degradation pathways of benzoate, benzene, and toluene merge to form the central metabolite benzoyl-CoA after which degradation continues via the benzoyl-CoA pathway. Elucidating mechanisms involved in anaerobic benzoate oxidation in the presence of energetically favorable carbon sources such as acetate contributes to understanding *G. daltonii* FRC-32’s metabolic plasticity and aromatic degradation via the benzoyl-CoA pathway.

Acetate, a two-carbon molecule, is readily converted to acetyl-CoA, which directly enters the citric acid cycle, facilitating efficient ATP production (13). In contrast, the aromatic compound benzoate requires multiple enzymatic steps for activation and conversion to intermediates compatible with central metabolic pathways, leading to higher energetic costs (14). Therefore, we hypothesize that acetate provides a more direct and energy-efficient pathway for cellular metabolism in *G. daltonii* FRC-32 compared to benzoate. It is crucial for strictly anaerobic microorganisms in dynamic environments to have the metabolic plasticity to facilitate the uptake and utilization of diverse substrates, particularly when additional carbon sources become available (15–18). Metabolic plasticity enables microbes to adjust to changing carbon source availability to maximize growth and metabolic activity (19, 20).

A metabolic strategy employed by microorganisms in environments with more than one carbon source available is to preferentially utilize the more energetically favorable carbon source before oxidizing other carbon sources (21–25). Carbon catabolite repression (CCR) is a ubiquitous regulatory system that facilitates the downregulation of degradation pathways of less energetically favorable carbon sources, thus promoting sequential carbon source utilization, leading to diauxic growth (26–28). CCR allows microorganisms to repress the utilization of aromatic hydrocarbons in the presence of high concentrations of more readily degradable carbon sources such as acetate or ethanol (15, 29–31). Repression of aromatic oxidation can be achieved by the downregulation of genes involved in each respective degradation pathway (31–34). For example, a study of the denitrifying bacterium *Azoarcus* sp. strain CIB (31) reported that the *bzdNOPQMSTUVWXYZA* genes, organized as a single catabolic operon and encoding the enzymes that facilitate benzoate catabolism, were repressed in the absence of benzoate.

Repression of aromatic oxidation pathways by CCR could diminish the potential of a microbe to facilitate bioremediation (35, 36). Therefore, an ideal candidate for clean-up of aromatic-contaminated environments requires the metabolic capacity to simultaneously oxidize multiple carbon sources (37, 38). Simultaneous carbon source oxidation is an alternative metabolic strategy employed by microorganisms inhabiting recalcitrant environments and leads to synchronous growth profiles (39), which was often observed in environments with low concentrations of carbon sources, as single carbon source utilization would not fulfill the energetic and thermodynamic demands of microbial communities under these oligotrophic conditions (40–42). Both simultaneous carbon source oxidation and CCR have been well reported (21, 43), but the metabolic mechanisms of anaerobic microorganisms in response to varying carbon source availability (particularly when aromatic or aliphatic carbon sources are available) have not been well studied (44).

The aim of this study was to elucidate potential mechanisms involved in anaerobic benzoate oxidation by *G. daltonii* FRC-32 in the presence of acetate. We focused on unraveling (i) benzoate oxidation mechanisms in *G. daltonii* FRC-32 cultures grown in the presence of acetate; (ii) regulation of genes involved in the benzoyl-CoA pathway during benzoate oxidation; and (iii) benzoate transport in *G. daltonii* FRC-32 whole cell lysates of cultures grown on benzoate in the presence of acetate.

## MATERIALS AND METHODS

### Culturing conditions

*G. daltonii* strain FRC-32 (DSM 22248; JCM 15807) (45) was grown under strictly anaerobic conditions as previously described (12, 46–48) with the following electron donors: benzoate (1 mM), acetate (2 or 5 mM), toluene (1 mM) or benzene (1 mM), and fumarate (10 mM) as an electron acceptor. Detailed cultivation conditions used in this study are listed in Table 1. *G. daltonii* FRC-32 cultures were adapted over six generations to grow on 1 mM benzoate + 2 mM acetate or 1 mM benzoate + 5 mM acetate to study mechanisms of benzoate oxidation when cultures were adapted to grow on two carbon sources. *G. daltonii* FRC-32 cultures were adapted over six generations to grow on 2 mM or 5 mM acetate and then spiked with 1 mM benzoate to study mechanisms of benzoate oxidation when cultures were adapted to grow on a readily available carbon source (acetate) when an aromatic carbon source (benzoate) became available. *G. daltonii* FRC-32 cultures were adapted over six generations to grow on 1 mM benzoate and then spiked with 2 mM acetate to study mechanisms of benzoate oxidation when cultures were adapted to grow on an aromatic carbon source (benzoate) when a readily available carbon source (acetate) became available.

**TABLE 1:** *G. daltonii* FRC-32 cultivation conditions with varying carbon sources and carbon source oxidation patterns

Carbon sources	Amendment	Carbon source oxidation pattern
1 mM benzoate +2 mM acetate^*a*^	N/A^*b*^	Simultaneous
1 mM benzoate +5 mM acetate^*a*^	N/A^*b*^	Simultaneous
2 mM acetate^*a*^	Spiked with 1 mM benzoate	Simultaneous
5 mM acetate^*a*^	Spiked with 1 mM benzoate	Sequential
1 mM benzoate^*a*^	Spiked with 2 mM acetate	Simultaneous

^*a*^ *G. daltonii* FRC-32 cultures were adapted over six generations to grow on these carbon sources.

^*b*^ N/A = not applicable.

### Analysis of substrates and metabolites

To measure the concentrations of acetate and benzoate, liquid media samples were anaerobically extracted from *G. daltonii* FRC-32 cultures and filtered via Nalgene 0.2 µm PTFE syringe filters (Nalgene Nunc, Rochester, NY) as previously described (12, 49). Cell filtrate was injected into a Dionex ICS-2000 Ion Chromatograph (IC) (Thermo Scientific Dionex, Canton, GA) equipped with a Dionex IonPac AS11-HC anionic resin column (Dionex, Sunnyvale, CA) and eluted with 5.6% 1 M KOH at a flow rate of 1.5 mL/min for 20 min.

### Analysis of benzoate accumulation in *G. daltonii* whole cell lysate

To analyze if benzoate accumulated intracellularly, whole cell lysates of 100 mL *G*. *daltonii* FRC-32 cultures grown on benzoate and acetate were prepared as previously described (12). Cells were pelleted, washed, resuspended in 500 µL deionized water, and subsequently transferred to a 0.8 mL glass serum bottle containing glass beads and 60 µL 1% SDS, and bead beaten for 1 min. 100 µL of lysate were filtered using a glass syringe and a 0.2 µm PTFE syringe filter (Nalgene Nunc, Rochester, NY) and were analyzed via IC.

### Total cellular protein analysis by SDS-PAGE

*G. daltonii* FRC-32 cells were harvested by centrifugation at room temperature for 20 min at 3,600 rpm. Cell pellets were resuspended in lysis solution and lysed for 6 min in boiling water. Upon lysis, whole protein lysates were resuspended in 1× NuPAGE sample buffer (Thermo Fisher Scientific, Wilmington, DE). Lysates were loaded onto a 10%/5% SDS-PAGE gel and migrated at 70 V through the stacking portion of the gel for 30 min before running at 100 V through the remainder of the gel for 2 h and 30 min. The gels were stained with Bio-Safe Coomassie G-250 stain (Bio-Rad, Hercules, CA) and de-stained with Coomassie destain solution. A more detailed method is described in Supplemental Material.

### Total RNA extraction

Total RNA was extracted from *G. daltonii* FRC-32 cultures as previously described by Chin *et al.* (50) with some modifications, including TM buffer (50 mM Tris-HCl [pH 7.0], 20 mM, and 140 MgCl_2_ in DEPC-treated water) instead of TPM buffer, cell disruption for 1 min at 2,500 rpm with a bead beater, without yeast tRNA and RNase inhibitor. The isolated RNA was treated with TURBO DNase (Life Technologies, Grand Island, NY). DNA removal was confirmed via PCR. RNA concentration and purity were determined using a NanoDrop 2000 UV-Vis Spectrophotometer (Thermo Fisher Scientific, Wilmington, DE).

### Primer design

Primers used in this study are listed in Table S1 and were synthesized by Integrated DNA Technologies (IDT) (San Jose, CA). All primers were designed specifically for this study based on the full genome sequence of *G. daltonii* strain FRC-32 (GenBank accession number NC_011979) using IDT PrimerQuest Tool (51) and manually screened according to the primer design rules (52). Primers were tested for primer dimer and hairpin formation, and validity was confirmed using IDT OligoAnalyzer Tool (51).

### Reverse transcription-polymerase chain reaction (RT-PCR)

cDNA synthesis was performed with gene-specific reverse primers, 0.5 µg total RNA, dNTP mix, RiboLock RNase inhibitor, and RevertAid RT reverse transcriptase (Thermo Fisher Scientific, Waltham, MA) incubated at 42°C for 60 min followed by enzyme inactivation at 70°C for 10 min. cDNA products were verified by PCR amplification via corresponding primer sets and visualized via gel electrophoresis.

### Quantitative real-time reverse transcription PCR (qRT-PCR)

Dilution series of purified RT-PCR amplicons, obtained with gene-specific primers, were used as calibration standards as described previously (49). All reactions were performed using SYBR Green PCR Master Mix (Life Technologies-Applied Biosystems, Grand Island, NY) and 20 pmol of each primer pair. The temperature profile was composed of an initial activation step at 50°C for 5 min and denaturation at 98°C for 40 s, followed by 40 cycles of denaturation at 98°C for 40 s, annealing at primer-specific temperature for 30 s, and elongation at 65°C for 30 s. Quantitative analysis was performed by the Applied Biosystems 7500 Real-Time PCR system (Life Technologies, Carlsbad, CA) with 7500 Real-Time PCR System Sequence Detection Software (Version 2.0.6). PCR product size and specificity were confirmed via agarose gel electrophoresis and Sanger sequencing.

### Identification of anaerobic benzoate oxidation genes

The genes *bamNOPQ* and *benK* were identified in the genome of *G. daltonii* strain FRC-32 (NC_011979) by using the *Basic Local Alignment Search Tool* (BLAST) pairwise alignment of the genes of *Geobacter metallireducens* (GenBank accession number NC_ 269799) and the genome of *G. daltonii* FRC-32 (53, 54).

### *In silico* protein structure and protein-ligand binding affinity prediction of putative aromatic transporter BenK

The molecular weight of BenK (Geob_0193) was estimated via Protein Molecular Weight, a JavaScript-based tool provided by the University of Alberta (55).

*In silico* modeling of BenK’s protein structure was performed via AlphaFold network (56). Accuracy and confidence values for structure predictions are provided as numerical confidence values, depicted on a pLDDT scale ranging from 0 to 100 (56). A more detailed method is described in Supplemental Material.

To predict the protein-ligand binding affinity of the putative aromatic transporter BenK, AutoDock/Vina 1.1.2 was used (57). Predicted binding sites were defined before docking runs were executed. Protein-ligand binding affinity predictions were represented as numerical values in kcal/mol (58). Root mean square deviation (RMSD) values for each protein-ligand prediction were provided. To differentiate the predicted binding affinities of the different tested substrates to BenK, an RMSD value difference of 2 Å was used as a cutoff. A more detailed method is described in Supplemental Material.

### Statistical analysis

Unpaired two-tailed Student *t*-tests were performed for statistical analysis at a probability level of *P* < 0.05. For growth (cell density) measurements, the results represented the means ± standard errors of triplicate OD_600_ determinations of each sample obtained from triplicate cultures. For analysis of substrates and metabolites, the results represented the means ± standard errors of triplicate IC determinations of each sample obtained from triplicate cultures. For gene expression analysis, the results represented the means ± standard errors of the triplicate qRT-PCR determinations of each cDNA sample obtained from triplicate cultures.

## RESULTS AND DISCUSSION

### Anaerobic oxidation of benzoate in the presence of acetate by *G. daltonii* FRC-32

**Fig 1:**
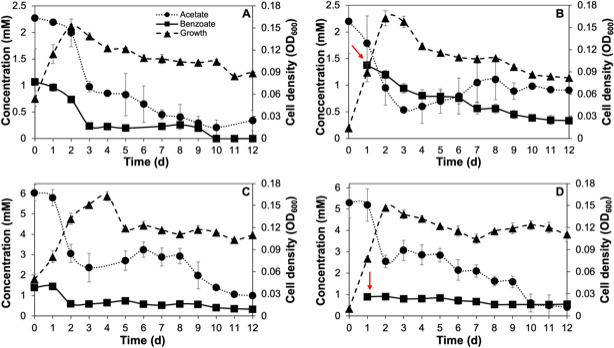
Growth characteristics and carbon source oxidation in *G. daltonii* FRC-32 cultures grown on various carbon sources. (A) Monoauxic growth and simultaneous carbon source oxidation in cultures on 1 mM benzoate + 2 mM acetate. (B) Monoauxic growth and simultaneous carbon source oxidation in cultures on 2 mM acetate cultures spiked with 1 mM benzoate. (C) Monoauxic growth and simultaneous carbon source oxidation in cultures on 1 mM benzoate + 5 mM acetate. (D) Diauxic growth and sequential carbon source oxidation in cultures on 5 mM acetate cultures spiked with 1 mM benzoate. Arrows indicate the addition of benzoate after 1-day incubation. The results represent the means ± standard errors of triplicate OD_600_ or IC determinations of each sample obtained from triplicate cultures.

*G. daltonii* FRC-32 cultures grown on 1 mM benzoate + 2 mM acetate and cultures on 2 mM acetate spiked with 1 mM benzoate reached maximum growth after 2 days (OD_600_ 0.152 and OD_600_ 0.163, respectively) (Fig. 1A and B). *G. daltonii* cultures grown on 1 mM benzoate + 5 mM acetate reached maximum growth after 4 days (OD_600_ 0.163) (Fig. 1C). *G. daltonii* cultures grown on 5 mM acetate spiked with 1 mM benzoate exhibited diauxic growth; in the first log phase, maximum growth was reached after 2 days (OD_600_ 0.147) and was followed by a lag phase before proceeding to a second log phase with maximum growth after 10 days (OD_600_ 0.124) (Fig. 1D).

Growth rates of *G. daltonii* FRC-32 cultures were determined for each condition. Growth rates of cultures grown on 1 mM benzoate + 5 mM acetate, 1 mM benzoate + 2 mM acetate, 5 mM acetate spiked with 1 mM benzoate, and 2 mM acetate spiked with 1 mM benzoate were higher than the rates of cultures grown on benzoate as sole carbon source (Fig. S1 and S2). Growth rates of cultures grown on 1 mM benzoate + 2 mM acetate were higher than the rates of cultures grown on 1 mM benzoate + 5 mM acetate. Growth rates of cultures on 2 mM acetate spiked with 1 mM benzoate were higher than the rates of the first log phase of cultures on 5 mM acetate spiked with 1 mM benzoate (Fig. S1 and S2).

Diauxic growth occurred only in *G. daltonii* FRC-32 cultures grown on 5 mM acetate spiked with 1 mM benzoate, whereas monoauxic growth occurred in all other cultures (Fig. 1 and Fig. S1). Diauxic growth coincided with sequential carbon source oxidation during which benzoate was initially not oxidized. Monoauxic growth and simultaneous carbon source utilization demonstrated that benzoate oxidation was not repressed. Several studies have reported that bacteria can repress the utilization of aromatic compounds (such as benzoate) in the presence of high concentrations of more energetically favorable carbon sources (such as acetate, malate, or ethanol) (15, 29–31, 59, 60). Da Silva *et al.* (59) found that addition of ethanol to BTEX-degrading aquifer columns decreased the efficacy of aromatic degradation by up to 77%. Barragan *et al.* (31) reported that addition of acetate, malate, and succinate to *Azoarcus* sp. strain CIB grown on benzoate caused severe catabolite repression after observing decreased benzoate consumption and decreased activity of benzoyl-CoA ligase. We therefore hypothesized that benzoate oxidation is not repressed in the presence of acetate.

In this study, we investigated whether *G. daltonii* FRC-32 performs sequential or simultaneous carbon source oxidation when grown on both acetate and benzoate as carbon sources (Table 1). In cultures grown on 1 mM benzoate + 2 mM acetate, both carbon sources were oxidized simultaneously within the first 3 days of incubation (Fig. 1A). In cultures grown on 2 mM acetate spiked with 1 mM benzoate, benzoate oxidation commenced shortly after the cultures were spiked with benzoate (Fig. 1B). In cultures grown on 1 mM benzoate + 5 mM acetate, both carbon sources were simultaneously oxidized within 2 days of incubation (Fig. 1C). Benzoate was not completely oxidized, indicating that carbon source concentrations possibly exceeded *G. daltonii* FRC-32’s metabolic capacity or requirements for growth. Similarly, in our previous study (12), we found that high carbon source concentrations could be metabolically unfavorable, leading to incomplete oxidation and accumulation of intermediates. In cultures grown on 5 mM acetate spiked with 1 mM benzoate, only acetate was oxidized within the first 2 days of cultivation. Benzoate oxidation began during the first decline phase, coinciding with a decrease in acetate concentration to approximately 2.5 mM (Fig. 1D). Neither acetate nor benzoate reached complete oxidation in cultures grown on 5 mM acetate spiked with 1 mM benzoate, indicating that carbon source concentrations exceeded *G. daltonii* FRC-32’s metabolic capacity or requirements for growth under limited conditions in batch culturing.

**Fig 2:**
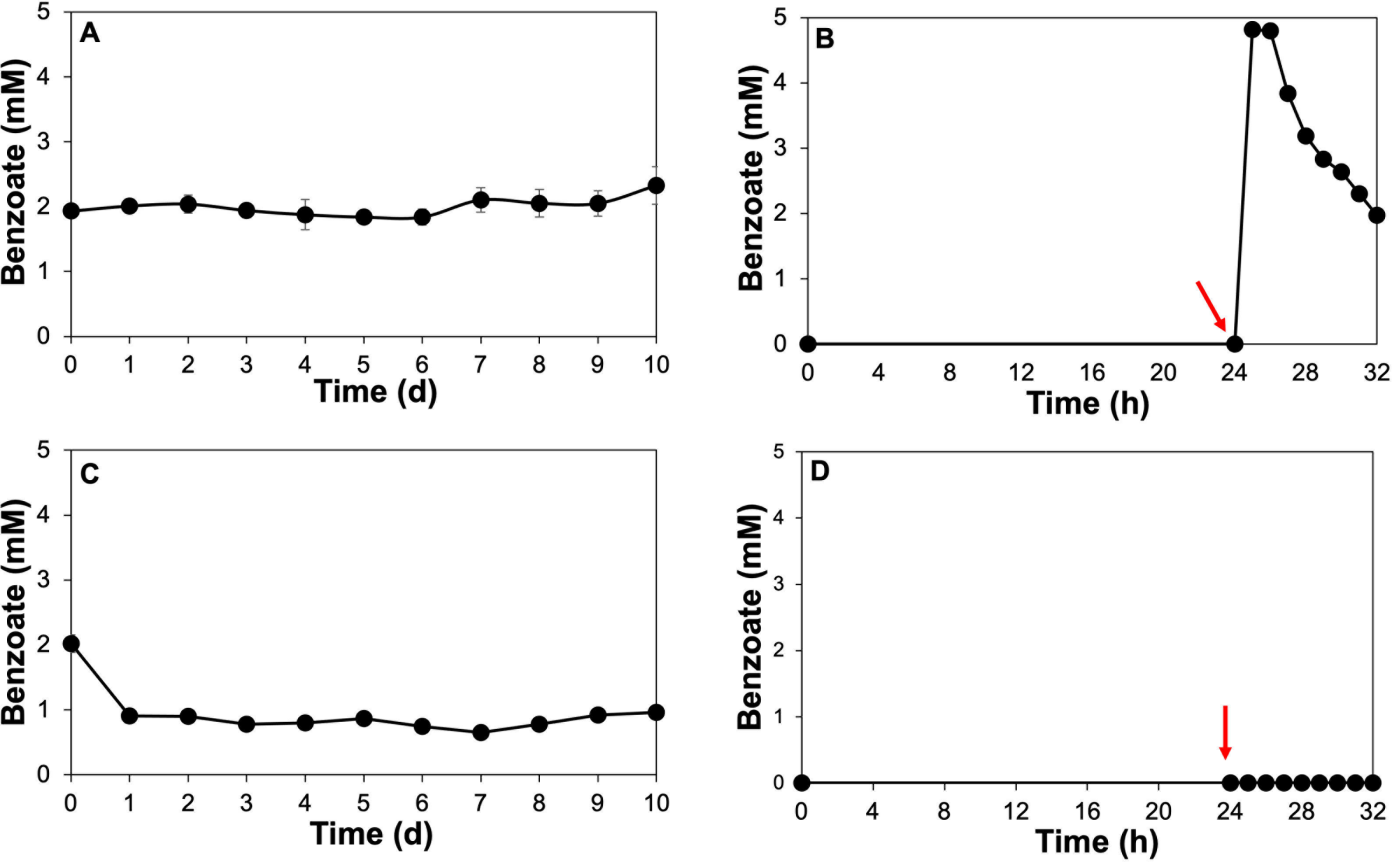
Benzoate accumulation in whole cell lysates of *G. daltonii* FRC-32 cultures grown on various carbon sources. (A) Cell lysate obtained from cultures grown on 1 mM benzoate + 2 mM acetate. (B) Cell lysate obtained from cultures grown on 2 mM acetate spiked with 1 mM benzoate. (C) Cell lysate obtained from cultures grown on 1 mM benzoate + 5 mM acetate. (D) Cell lysate obtained from cultures grown on 5 mM acetate spiked with 1 mM benzoate. Arrows indicate the addition of benzoate after 1-day incubation. The results represent the means ± standard errors of triplicate IC determinations of each sample obtained from triplicate cultures.

We examined intracellular benzoate accumulation to elucidate benzoate transport mechanism into the cell, as the presence of benzoate intracellularly could induce expression of the benzoyl-CoA pathway. In the whole cell lysates obtained from cultures grown on 1 mM benzoate + 2 mM acetate, intracellular benzoate concentration increased at the end of the incubation period (day 7 through 10) (Fig. 2A). The highest intracellular benzoate accumulation was observed on day 10, coinciding with extracellular benzoate depletion (Fig. 1A). Benzoate, the electron donor, may accumulate intracellularly due to limited availability of terminal electron acceptor causing a halt in electron transfer, depletion of other nutrients, or accumulation of metabolic waste products. In the cell lysates obtained from cultures grown on 1 mM benzoate + 5 mM acetate, intracellular benzoate concentrations of *ca*. 1 mM were observed (Fig. 2C). Benzoate accumulation was observed in the cell lysate obtained from cultures grown on 2 mM acetate spiked with 1 mM benzoate 1 h after the cultures were spiked with benzoate (Fig. 2B). This suggested that benzoate transport commenced immediately after the cultures were spiked with 1 mM benzoate, indicating that lower concentrations of 2 mM acetate facilitated *G. daltonii* FRC-32’s readiness to transport and oxidize benzoate once it was available. No intracellular benzoate accumulation was detected in the cell lysate obtained from cultures grown on 5 mM acetate spiked with 1 mM benzoate (Fig. 2D). This may have occurred because it was not metabolically favorable for *G. daltonii* to transport and oxidize another carbon source or that 5 mM acetate prevented benzoate transport, indicating that high concentration of acetate did not facilitate *G. daltonii* FRC-32’s readiness to commence benzoate oxidation when benzoate became available as a carbon source.

In summary, our results demonstrated simultaneous benzoate and acetate oxidation in *G. daltonii* FRC-32 cultures grown on 1 mM benzoate + 5 mM acetate, 1 mM benzoate + 2 mM acetate, and 2 mM acetate spiked with 1 mM benzoate (Fig. 1; Table 1). Sequential carbon source oxidation was only observed in cultures grown on 5 mM acetate spiked with 1 mM benzoate (Fig. 1D; Table 1) indicating contingency on two factors: the concentration of acetate and the specific cultivation condition. Sequential oxidation was only observed when cultures were adapted over six generations to grow on 5 mM acetate without benzoate and later spiked with 1 mM benzoate as an additional carbon source. This indicated that 5 mM acetate as sole carbon source was sufficient to meet the metabolic requirements of *G. daltonii* FRC-32 and that benzoate oxidation only commenced when acetate was not sufficiently available to fulfill *G. daltonii*’s metabolic requirement. Benzoate oxidation commenced immediately in cultures grown on 2 mM acetate spiked with 1 mM benzoate (Fig. 1B) and commenced in cultures grown on 5 mM acetate spiked with 1 mM benzoate when acetate concentration reached *ca*. 2.5 mM (Fig. 1D), indicating an acetate concentration-dependent threshold below which benzoate transport and oxidation occurred. Furthermore, there was an inverse correlation between acetate availability and the extent of benzoate oxidation; in cultures grown at lower acetate concentration (2 mM), benzoate was almost completely oxidized, and at a faster rate. This indicated that the availability of acetate at lower concentrations was more conducive to benzoate oxidation in *G. daltonii* FRC-32. However, *G. daltonii* FRC-32, unlike other anaerobic aromatic-degrading bacteria (29–31, 56), including its close relative *G. metallireducens* (15), could simultaneously utilize benzoate and the energetically more favorable carbon source acetate, even when both carbon sources were available at high concentrations.

**Fig 3:**
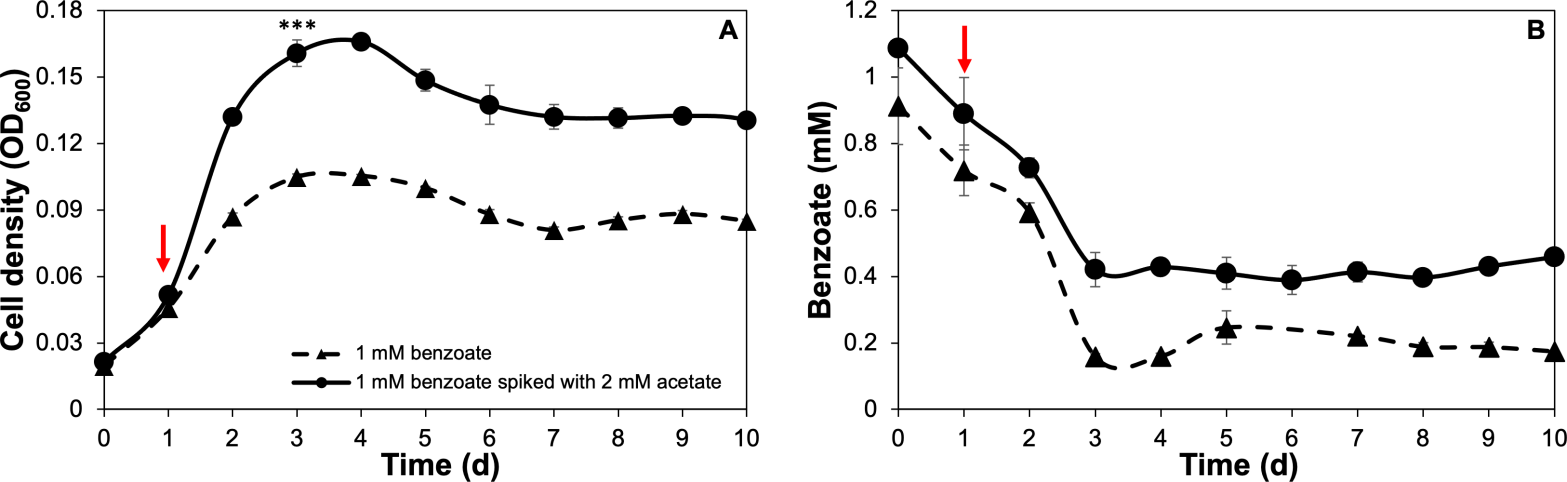
*G. daltonii* FRC-32’s growth was enhanced in cultures grown on 1 mM benzoate spiked with 2 mM acetate. (A) Growth patterns of *G. Daltonii*. (B) Benzoate oxidation in cultures grown on 1 mM benzoate as a sole carbon source or on 1 mM benzoate spiked with 2 mM acetate. Arrows indicate the addition of acetate after 1-day incubation. The results represent the means ± standard errors of triplicate OD_600_ or IC determinations of each sample obtained from triplicate cultures (***P > 0.0005 as determined by student’s *t*-test). Significant difference compared to cell density during growth on 1 mM benzoate is indicated by asterisks.

**Fig 4:**
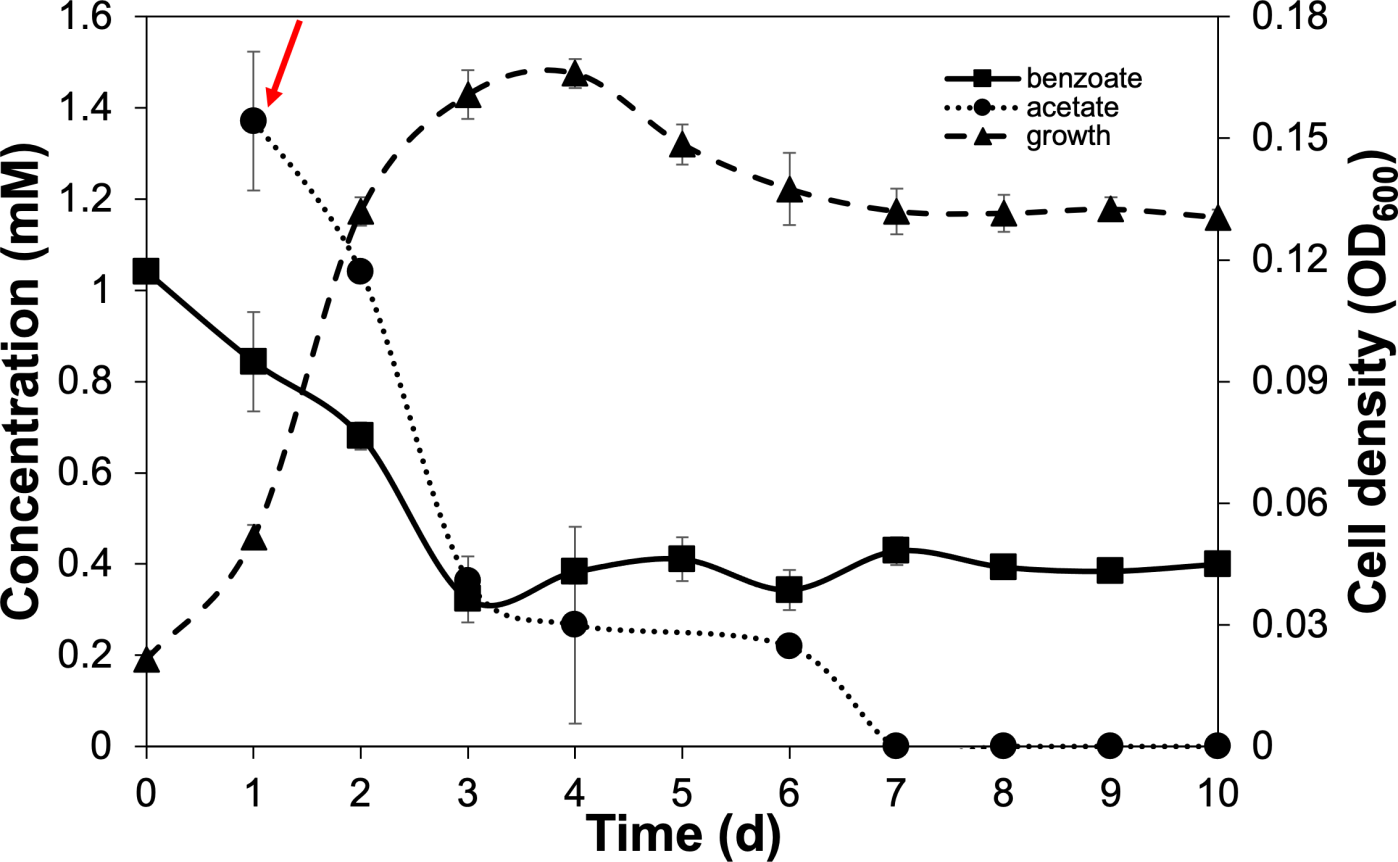
Monoauxic growth and simultaneous carbon source oxidation in *G. daltonii* FRC-32 cultures grown on 1 mM benzoate spiked with 2 mM acetate. Carbon source oxidation in cultures on 1 mM benzoate spiked with 2 mM acetate. Arrow indicates addition of acetate after 1-day incubation. The results represent the means ± standard errors of triplicate OD_600_ or IC determinations of each sample obtained from triplicate cultures.

Benzoate oxidation was further elucidated after *G. daltonii* FRC-32 cultures were adapted to grow on benzoate as an aromatic carbon source with a more energetically favorable carbon source becoming available later: cultures were adapted to grow on 1 mM benzoate and spiked with 2 mM acetate. The availability of an additional carbon source (such as acetate) can increase the number of microorganisms that perform bioremediation, a form of biostimulation (61). *G. daltonii* cultures grown on 1 mM benzoate spiked with 2 mM acetate reached maximum growth after 4 days (OD_600_ 0.166), which was significantly higher than maximum growth of cultures grown on benzoate as sole carbon source (OD_600_ 0.105) (Fig. 3A). Benzoate was rapidly oxidized within the first 3 days in cultures grown on 1 mM benzoate spiked with 2 mM acetate (Fig. 3B and 4). Acetate oxidation commenced immediately after cultures grown on 1 mM benzoate were spiked with acetate (Fig. 4). Benzoate was not completely oxidized (Fig. 3B and 4), possibly due to cultures on 1 mM benzoate spiked with 2 mM acetate reaching significantly higher maximum growth than cultures grown on 1 mM benzoate. Nutrient depletion or accumulation of metabolic end products, associated with high cell densities, have been reported to inhibit carbon source oxidation. For example, Pham *et al.* (62) reported that high cell densities and accumulation of metabolic by-products such as ethanol had an inhibitory effect on maximum respiration and carbon source oxidation in *Saccharomyces cerevisiae* grown in batch culture experiments.

Our results demonstrated that in *G. daltonii* FRC-32 cultures grown on 1 mM benzoate spiked with 2 mM acetate, both carbon sources were simultaneously oxidized, but benzoate was not completely oxidized. This suggested that although benzoate oxidation was not enhanced, acetate did not prevent benzoate oxidation in cultures grown on 1 mM benzoate spiked with 2 mM acetate. This finding indicates that differential oxidation of benzoate by *G. daltonii* FRC-32 may be regulated in response to dynamic environmental conditions.

### Pre-cultivation conditions with varying benzoate and acetate availability determined the growth characteristics of benzoate-oxidizing *G. daltonii* FRC-32 cultures

To elucidate *G. daltonii* FRC-32’s strategies to adapt their metabolism in response to changing environmental conditions, *G. daltonii* was cultured on 1 mM benzoate as sole carbon source using inocula from the following pre-cultivation conditions: cultures grown on 5 mM acetate + 1 mM benzoate, cultures grown on 2 mM acetate + 1 mM benzoate, cultures grown on 5 mM acetate spiked with 1 mM benzoate, and cultures grown on 2 mM acetate spiked with 1 mM benzoate, respectively.

**Fig 5:**
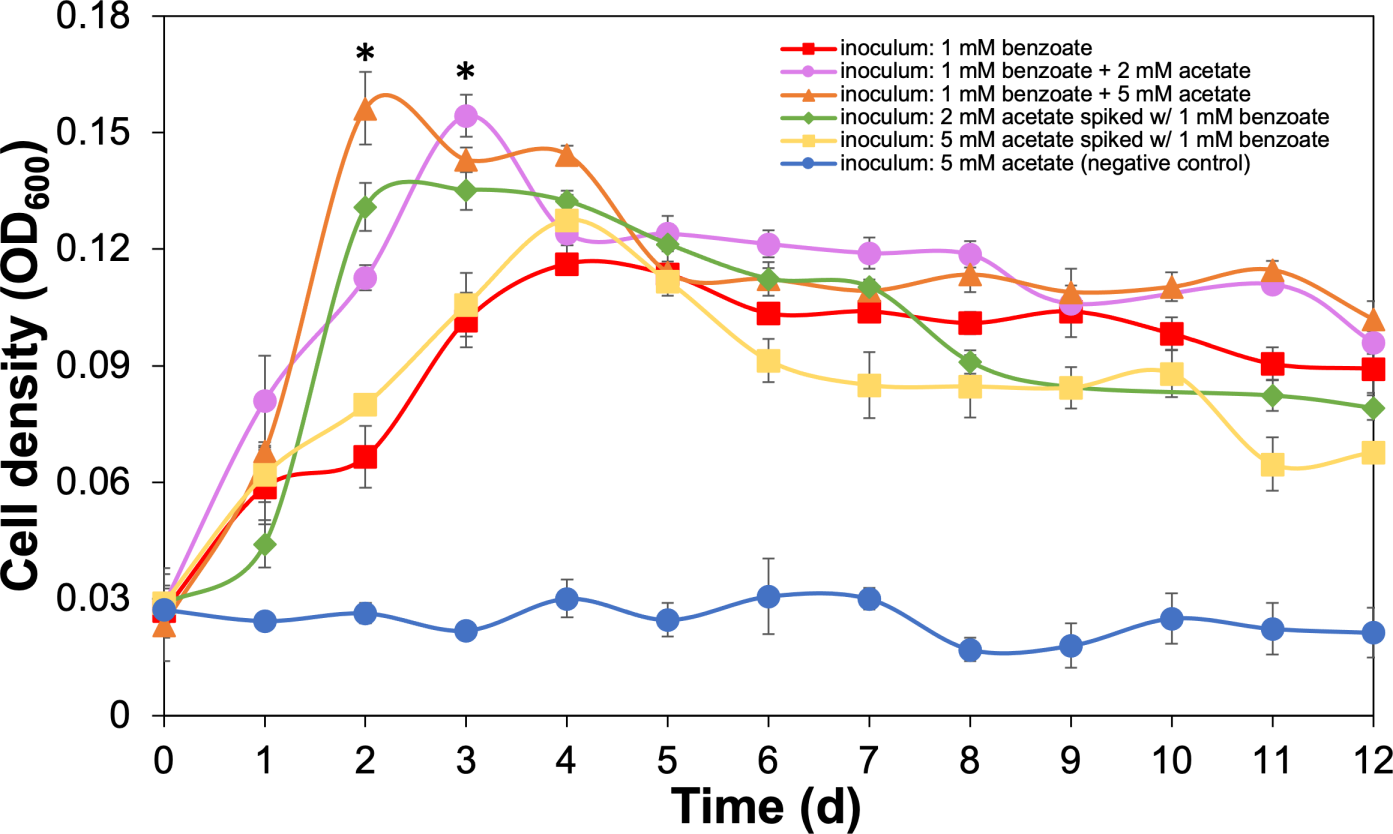
Effect of pre-cultivation conditions on growth characteristics of benzoate-oxidizing *G. daltonii* FRC-32 cultures. Benzoate-oxidizing cultures were inoculated from various cultures that were adapted to varying benzoate and/or acetate availability. The results represent the means ± standard errors of triplicate OD_600_ determinations of each sample obtained from triplicate cultures. (*P > 0.05 as determined by student’s *t*-test). Significant difference compared with cell density in benzoate-oxidizing cultures inoculated with cultures grown on 2 mM acetate spiked with 1 mM benzoate or 5 mM acetate spiked with 1 mM benzoate is indicated by asterisks.

Benzoate-oxidizing *G. daltonii* FRC-32 cultures were inoculated with cultures pre-grown on benzoate + acetate (1 mM benzoate + 2 mM acetate or 1 mM benzoate + 5 mM acetate) and reached a significantly higher cell density than the ones that were inoculated with cultures pre-grown on acetate spiked with benzoate (2 mM acetate spiked with 1 mM benzoate or 5 mM acetate spiked with 1 mM benzoate) (Fig. 5). *G. daltonii* FRC-32 cultures grown on benzoate that were inoculated with cultures pre-grown on 5 mM acetate spiked with 1 mM benzoate reached the lowest growth and grew at the slowest rate compared with the cultures grown on benzoate that were inoculated from cultures pre-grown on 1 mM benzoate +2 mM acetate, or 1 mM benzoate + 5 mM acetate, or 2 mM acetate spiked with 1 mM benzoate. Cultures grown on benzoate that were inoculated with cultures pre-grown on acetate did not grow. Cultures pre-grown on acetate were not adapted to metabolizing benzoate, indicating that CCR of benzoate oxidation occurred in cultures grown on acetate and likely making metabolic adaptation in response to the presence of an additional carbon source (benzoate) unfavorable and preventing rapid modulation of genes that would facilitate the sudden switch to benzoate oxidation.

These findings demonstrated that the carbon sources used to pre-cultivate the inoculum determined the growth characteristics of *G. daltonii* FRC-32 cultures grown on benzoate. Specifically, all cultures grown on benzoate that were inoculated with cultures pre-grown on varying availability of benzoate and acetate were able to metabolically adapt to changing carbon source availability. In natural environments, microorganisms are exposed to various carbon sources, demanding metabolic plasticity of the microorganism to switch from the utilization of one carbon source to another, once the preferred carbon source is depleted (63–65). Microorganisms capable of metabolic plasticity are thought to grow rapidly without a lag phase in response to carbon source amendments (66).

### Benzoate transport in *G. daltonii* FRC-32 cultures grown on acetate and benzoate

To elucidate the dynamics of anaerobic benzoate oxidation in *G. daltonii* FRC-32, benzoate transport mechanisms were investigated. The gene *benK* (Geob_0193), encoding the putative benzoate transporter BenK, is located adjacent to the benzoate degradation gene clusters (Geob_0095-Geob_0100 and Geob_0200-Geob_0235) (Table S2; Fig. S4). Close genomic proximity of the gene *benK* to the benzoate degradation gene clusters indicated that BenK facilitates benzoate transport. Previous studies reported that genes encoding transport proteins and degradation enzymes for a specific substrate were often co-located within the genome (67, 68).

**Fig 6:**
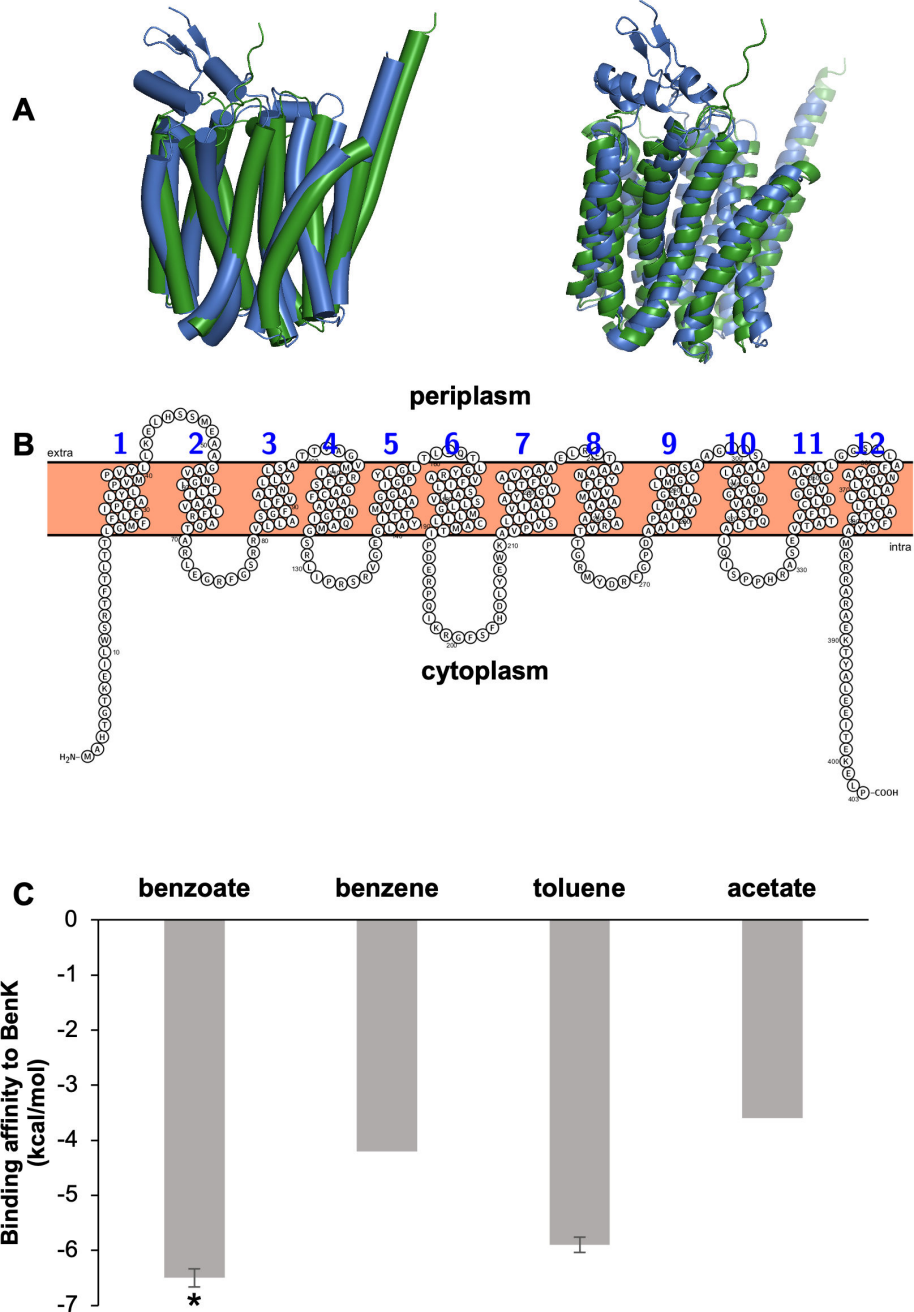
Structure and protein-ligand-binding affinity prediction of the putative benzoate transporter BenK in *G. daltonii* FRC-32. (A) *In silico* structure prediction of BenK shows the presence of transmembrane regions creating a central transport channel (left). Structural alignment prediction of BenK compared with *P. putida* CSV86’s BenK (green: *G. daltonii* FRC-32; blue: *P. putida*) (right). (B) Protein topology prediction for BenK reveals the presence of 12 transmembrane regions. (C) *In silico* prediction of protein-ligand binding affinity of aromatic compounds to BenK. Protein-ligand binding affinity predictions were performed in triplicates. The results are represented as the means ± standard errors (*P > 0.05; as determined by Student’s *t*-test). Significant difference compared to binding affinity of BenK to acetate, toluene, and benzene is indicated by an asterisk.

The three-dimensional structure prediction of BenK revealed the presence of 12 transmembrane regions that are arranged in a barrel-like manner creating a central transport channel. The same structure was reported for other benzoate transporter proteins including BenK in *Pseudomonas putida* CSV86 (67, 69–76) (Fig. 6A and B). Microbial benzoate transport has been most thoroughly investigated (*in vivo* and *in vitro*) in *P. putida* CSV86 (67). *In silico* structural alignment of BenK of both *G. daltonii* FRC-32 and *P. putida* CSV86 (67) revealed high structural similarity and spatial arrangement (Fig. 6A). *In silico* binding affinity predictions (57) suggested that BenK may bind benzoate, toluene, benzene, and acetate with an affinity of −6.5 kcal/mol, −5.9 kcal/mol, −4.2 kcal/mol, and −3.6 kcal/mol, respectively (Fig. 6C).

**Fig 7:**
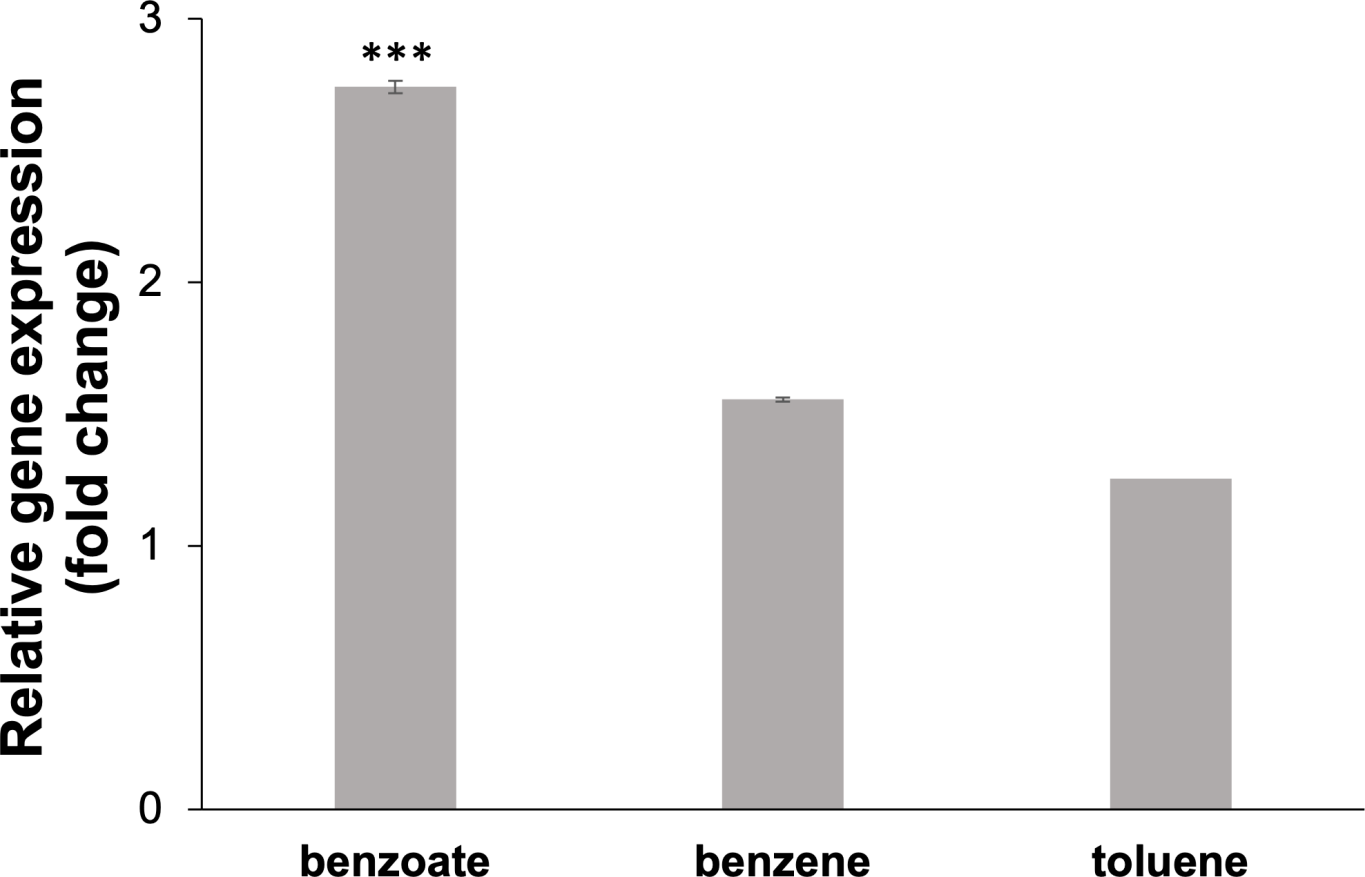
Relative expression levels of putative benzoate transporter gene *benK* (Geob_0193) in *G. daltonii* FRC-32 cultures grown on different aromatic carbon sources. Transcript levels for *benK* were normalized to transcript levels for housekeeping gene *recA*. The fold change is relative to expression levels of *benK* in cultures grown on acetate as a negative control. The results represent the means ± standard errors of the triplicate qRT-PCR determinations of each cDNA sample obtained from triplicate cultures (***P > 0.0005; as determined by student’s *t*-test). Significant difference compared with expression during growth on benzene and toluene is indicated by asterisks.

To elucidate specificity of the gene *benK* for aromatic carbon sources in *G. daltonii* FRC-32 *in vitro*, transcript levels for the gene *benK* were quantified in cultures grown on different aromatic compounds (Fig. 7). Choudhary *et al.* (67, 68) reported that the expression of the genes *benK* and *benE*, encoding benzoate transporters, were induced in *P. putida* CSV86 in the presence of benzoate. In our study, transcript levels for the gene *benK* in *G. daltonii* FRC-32 cultures were significantly higher during benzoate oxidation (3-fold) than during benzene (1.5-fold) or toluene (1.2-fold) oxidation (Fig. 7). These findings suggested that expression of the gene *benK* was induced by the presence of its predicted ligand, benzoate, further supporting our hypothesis that BenK may function as benzoate transporter in *G. daltonii* FRC-32.

**Fig 8:**
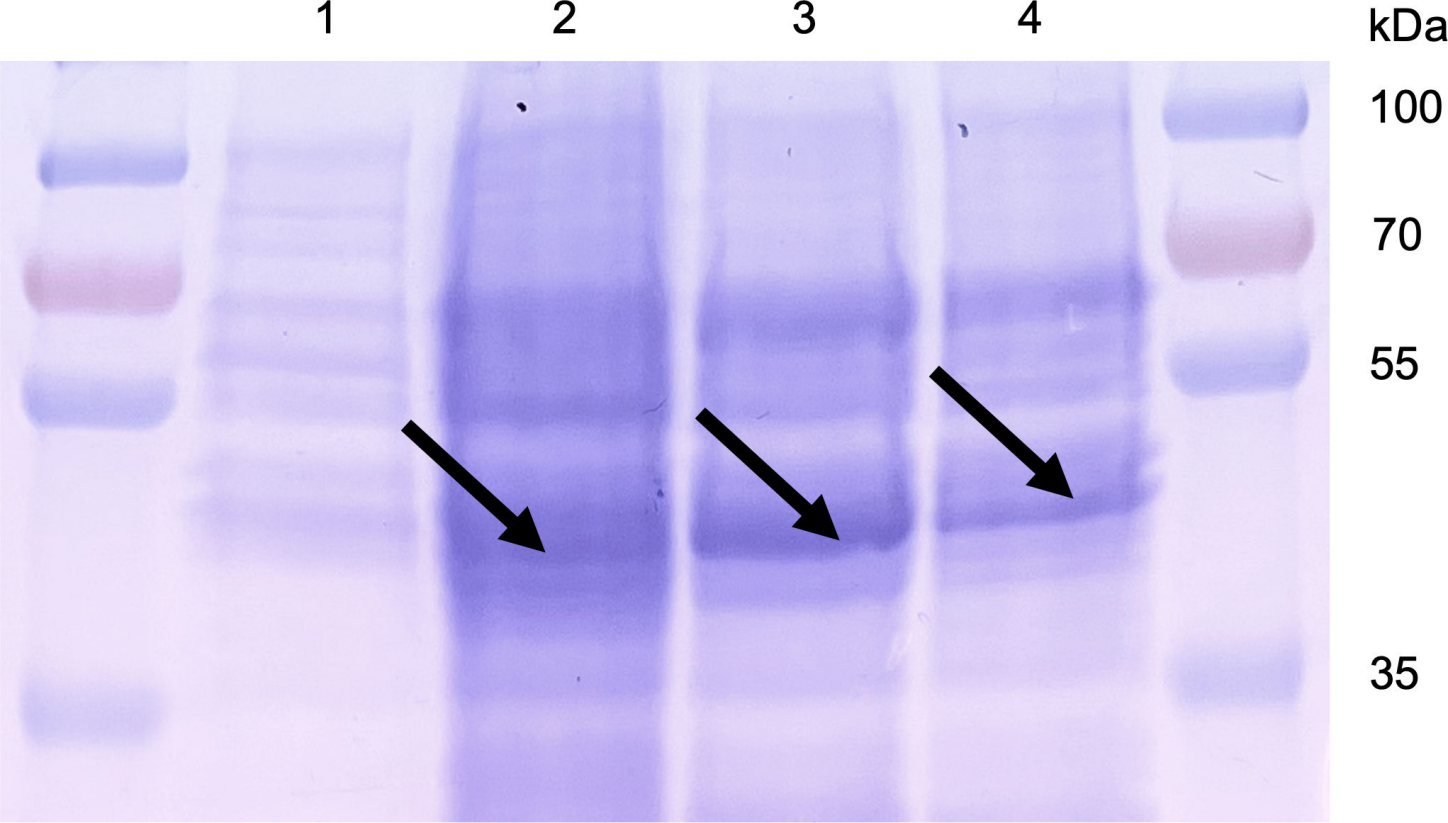
SDS-PAGE image showing the band indicative of putative benzoate transporter BenK in whole cell lysates of *G. daltonii* FRC-32 cultures grown on various aromatic compounds. *Lane 1:* cells grown on 5 mM acetate as the sole carbon source. *Lane 2:* cells grown on 1 mM benzoate as sole carbon source. *Lane 3:* cells grown on 1 mM toluene as the sole carbon source. *Lane 4:* cells grown on 1 mM benzene. Arrows indicate the location of the putative benzoate transporter BenK, *ca*. 43 kDa.

Total cellular protein profiles of whole cell lysates obtained from *G. daltonii* FRC-32 cultures grown on 1 mM benzoate, 1 mM benzene, 1 mM toluene, or 5 mM acetate were visualized via SDS-PAGE (Fig. 8) and showed the presence of a band corresponding to the predicted size of BenK (*ca*. 43 kDa) (55) (Table S3) during benzoate, toluene, or benzene oxidation. No band corresponding to the size of BenK (55) was observed in whole cell lysates extracted from *G. daltonii* FRC-32 cultures grown on acetate as the sole carbon source. This finding indicated that the expression of BenK was potentially induced by the presence of aromatic compounds and highlights its potential role in the metabolism of aromatic substrates in *G. daltonii* FRC-32 (Fig. 8). In summary, our results suggested that BenK facilitated benzoate transport in *G. daltonii* FRC-32 (Fig. 6 to 8).

**Fig 9:**
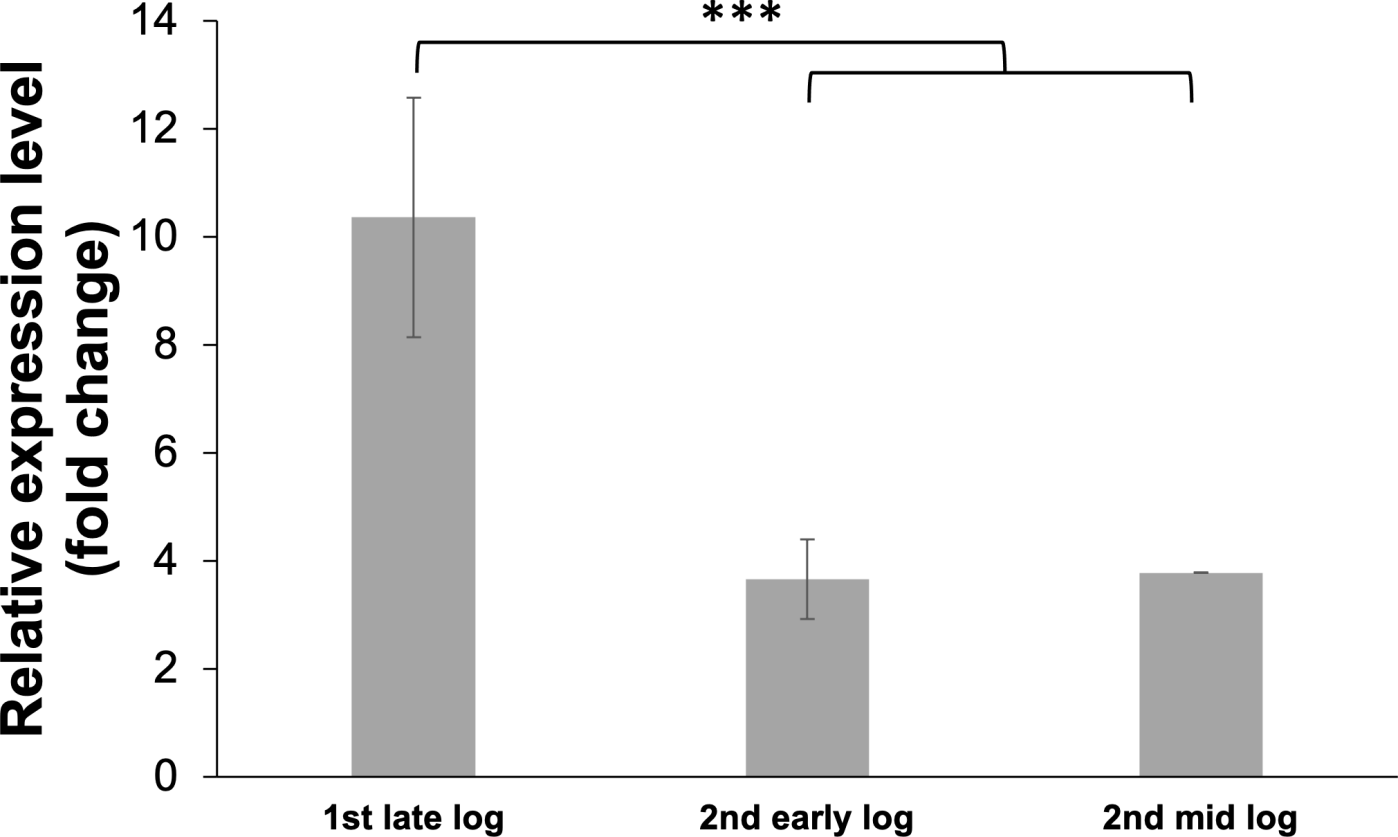
Relative expression levels of putative aromatic transporter gene *benK* (Geob_0193) in different growth phases of *G. daltonii* FRC-32 cultures grown on 5 mM acetate and spiked with 1 mM benzoate. Transcript levels for *benK* were normalized to transcript levels for housekeeping gene *recA*. The fold change is relative to expression levels of *benK* in cultures grown on acetate as a negative control. The results represent the means ± standard errors of the triplicate qRT-PCR determinations of each cDNA sample obtained from triplicate cultures (***P > 0.0005; as determined by student’s *t*-test). Significant difference compared with expression during the second early and mid-log phases is indicated by asterisks.

**Fig 10:**
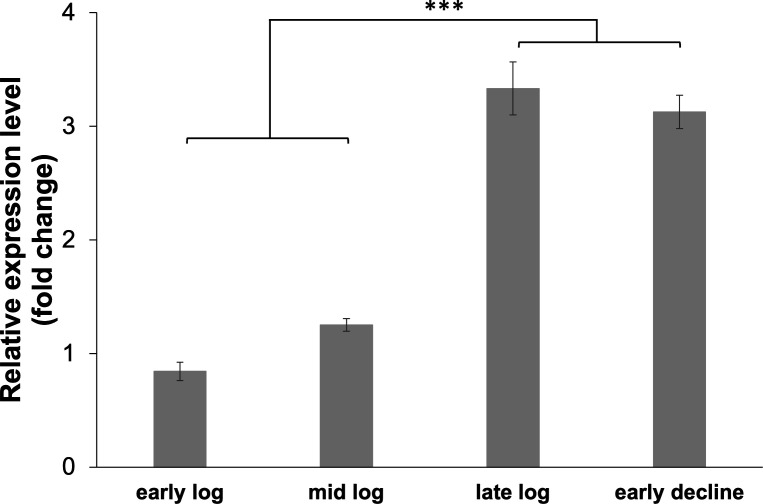
Relative expression levels of putative aromatic transporter gene *benK* (Geob_0193) in different growth phases of *G. daltonii* FRC-32 cultures grown on 1 mM benzoate + 5 mM acetate. Transcript levels for *benK* were normalized to transcript levels for housekeeping gene *recA*. The fold change is relative to expression levels of *benK* in cultures grown on acetate as a negative control. The results represent the means ± standard errors of the triplicate qRT-PCR determinations of each cDNA sample obtained from triplicate cultures (***P > 0.0005; as determined by student’s *t*-test). Significant difference compared with expression during the early and mid-log phases is indicated by asterisks.

Relative expression levels of the gene *benK* in cultures grown on benzoate and acetate were determined to elucidate the role of benzoate transport during differential benzoate oxidation (Fig. 9). Fold change was determined relative to expression levels of the gene *benK* grown on acetate. In cultures grown on 5 mM acetate spiked with 1 mM benzoate, expression of the gene *benK* was significantly highest during the first late log phase (10-fold) (Fig. 9). Expression was significantly upregulated in the second early log (3.5-fold) and second mid-log phases (3.5-fold) (Fig. 9). These findings demonstrated upregulation of the gene *benK* in *G. daltonii* FRC-32 cultures after the cultures were spiked with benzoate. Transcript levels for the gene *benK* in *G. daltonii* FRC-32 cultures grown on 5 mM acetate + 1 mM benzoate increased from early- and mid-log (both 1-fold) to late-log and early decline phases (both 3-fold) (Fig. 10).

Expression of putative benzoate transporter BenK was analyzed via SDS-PAGE in total cellular protein profiles of *G. daltonii* FRC-32 cultures grown on acetate and benzoate and revealed the presence of a band corresponding to the predicted size of BenK (*ca*. 43 kDa) (55) (Table S3; Fig. S3). This result suggested that BenK was expressed in all tested *G. daltonii* FRC-32 cultures grown on acetate and benzoate as carbon sources and indicated that BenK facilitated benzoate transport inside the cell, where benzoate could induce expression of the benzoyl-CoA pathway. Similarly, a study by Marozava *et al.* (15) reported that expression of the gene *benK* and benzoate transport, leading to benzoate entering the cell, could induce expression of the benzoyl-CoA pathway and explain the lack of acetate-induced CCR in *G. metallireducens* cultures grown on acetate and benzoate (15).

### Regulation of *bamNOPQ* in *G. daltonii* FRC-32 cultures grown on acetate and benzoate

**Fig 11:**
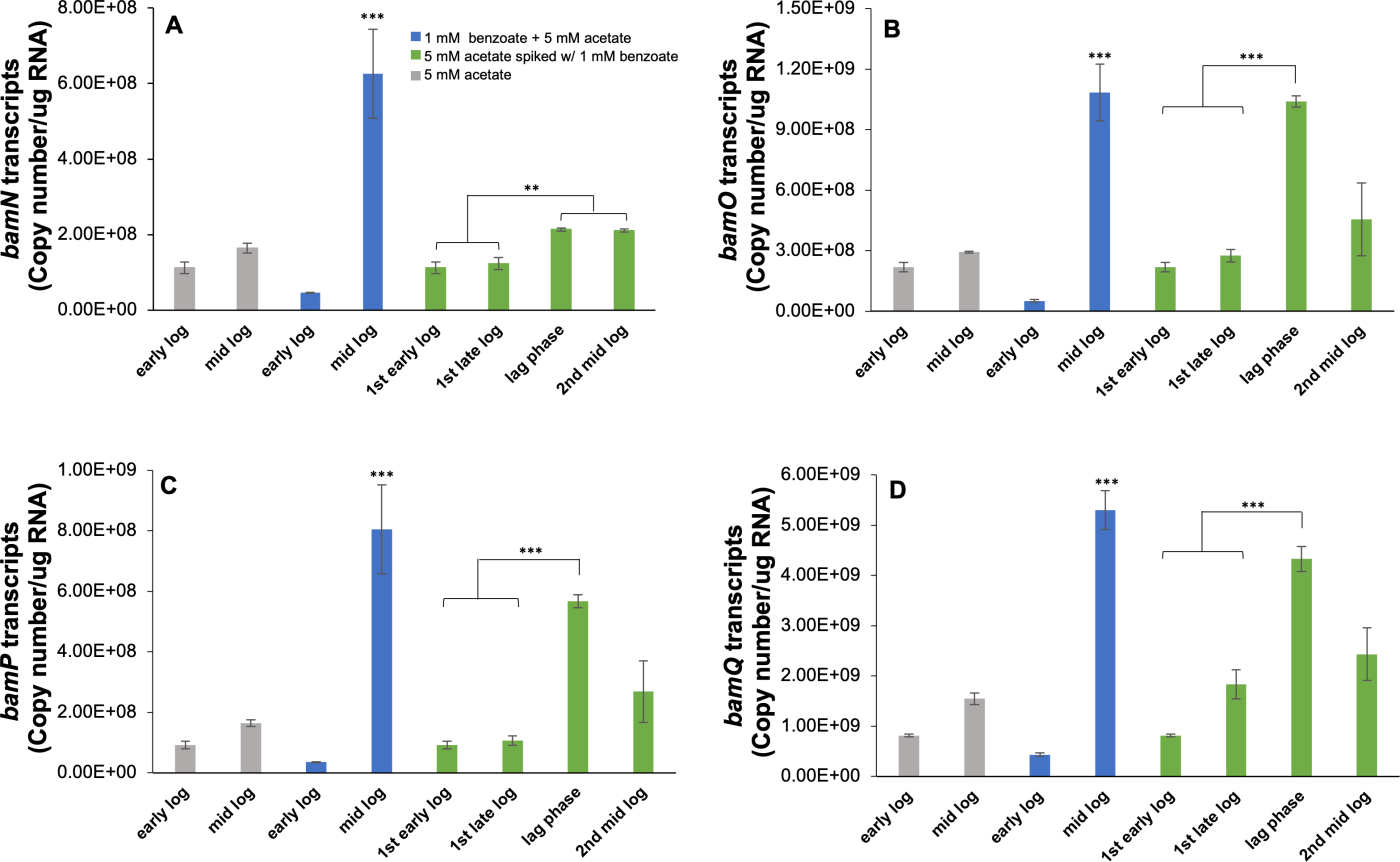
Transcript levels for genes *bamNOPQ* in *G. daltonii* FRC-32 cultures grown on 1 mM benzoate + 5 mM acetate, 5 mM acetate spiked with 1 mM benzoate and 5 mM acetate. (A) Transcript levels for gene *bamN*, encoding thiolase. (B) and (C) Transcript levels for genes *bamO* and *bamP,* encoding the electron transfer flavoprotein subunits beta and alpha, which facilitate electron transfer for the strictly ETF-coupled benzoate degradation protein BamM (glutaryl-coenzyme A dehydrogenase). (D) Transcript levels for gene *bamQ,* encoding 6-hydroxycyclohex-1-ene-1-carbonyl-CoA dehydrogenase. The results represent the means ± standard errors of the triplicate qRT-PCR determinations of each cDNA sample obtained from triplicate cultures (***P > 0.0005, **P > 0.005; as determined by Student’s *t*-test). Significant difference compared to transcript levels during the early and late-log phases is indicated by asterisks.

To elucidate the expression of genes involved in anaerobic benzoate oxidation by *G. daltonii* FRC-32 during either simultaneous or sequential carbon source oxidation, transcript levels for the genes *bamNOPQ* were quantified in *G. daltonii* cultures grown on 5 mM acetate + 1 mM benzoate; in cultures grown on 5 mM acetate spiked with 1 mM benzoate; and in cultures grown on acetate as sole carbon source (as negative control) (Fig. 11). The gene *bamN* (Geob_0100) encodes thiolase, which was reported to be active during anaerobic benzoate oxidation by facilitating formation of acetyl-CoA (15) (Table S2). The genes *bamO* and *bamP* (Geob_0095 and Geob_0096) encode the electron transfer flavoprotein (ETF) subunits beta and alpha, respectively, which facilitate electron transfer for the strictly ETF-coupled benzoate degradation protein BamM (glutaryl-coenzyme A dehydrogenase) (Table S2). The gene *bamQ* (Geob_0097) encodes 6-hydroxycyclohex-1-ene-1-carbonyl-CoA dehydrogenase, which is a downstream benzoate degradation protein (33, 77–81) (Table S2). Downregulation of genes *bamNOPQ* was expected during growth on acetate as BamNOPQ are not associated with acetate oxidation. Carmona *et al.* (82) reported that a gene *bamN*, closely associated with the benzoate degradation gene cluster, was the gene with the greatest increase in transcript levels during the growth of *G. metallireducens* on benzoate. A study by Estelmann and Boll (77) characterized BamOP, which functioned as ETF in glutaryl-CoA oxidation/crotonyl-CoA reduction during anaerobic benzoate oxidation by *G. metallireducens*. Laempe *et al.* (80) reported that BamQ facilitated conversion from 6-hydroxycyclohex-1-ene-1-carbonyl-CoA and NAD^+^ to 6-oxocyclohex-1-ene-1-carbonyl-CoA and NADH during anaerobic benzoate oxidation in *Thauera aromatica*. In *G. daltonii* FRC-32 cultures grown on acetate, transcript levels for the genes *bamNOPQ* were significantly lower compared with mid-log phases of cultures on 5 mM acetate + 1 mM benzoate and lag phases of cultures on 5 mM acetate spiked with 1 mM benzoate and lower (although not significantly) compared with second mid-log phases of cultures on 5 mM acetate spiked with 1 mM benzoate (Fig. 11).

In cultures grown on 5 mM acetate + 1 mM benzoate, transcript levels for genes *bamNOPQ* in early log phase were similar to ones demonstrated during growth on acetate as sole carbon source (Fig. 11). Transcript levels for genes *bamNOPQ* in mid log were significantly higher than those of cultures grown on acetate only, suggesting that acetate did not induce repression. These results corresponded to our findings of simultaneous carbon source oxidation in cultures grown with 5 mM acetate + 1 mM benzoate.

Transcript levels for genes *bamNOPQ* in cultures grown on 5 mM acetate spiked with 1 mM benzoate during the first early log and first late log phase were similar to ones for genes *bamNOPQ* in cultures grown on acetate as the sole carbon source (Fig. 11). However, transcript levels in the lag phases were significantly higher than ones in first early and first late log phases, suggesting that acetate repressed expression of genes *bamNOPQ* in the first log phase, but not in the lag phase, thus leading to sequential carbon source oxidation. Transcript levels in the second mid-log phases were higher (although not significantly) than the ones in the first early and first late log phases. Similar to our observations, a high expression of BamNOPQ during preferential acetate utilization in *G. metallireducens* cultures with acetate and benzoate as carbon sources available was reported by Marozava *et al.* (15).

In summary, higher transcript levels for the genes *bamNOPQ* were observed during simultaneous carbon source oxidation in cultures grown on 1 mM benzoate + 5 mM acetate as well as during sequential carbon source oxidation in the second log phase of cultures grown on 5 mM acetate spiked with 1 mM benzoate, indicating that varying carbon source availability led to differential gene expression of genes *bamNOPQ*, ultimately facilitating differential benzoate oxidation.

### Conclusions

This study investigated anaerobic benzoate oxidation via the benzoyl-CoA pathway in the presence of acetate by *G. daltonii* strain FRC-32. Our findings demonstrated that *G. daltonii* FRC-32 can oxidize benzoate differentially in response to varying carbon source availability, by either simultaneous or sequential carbon source oxidation. Sequential carbon source oxidation is widely accepted to be attributed to CCR (83). However, our findings suggested that the repression of benzoate oxidation in *G. daltonii* FRC-32 occurred as an adaptive regulatory response to specific carbon source availability and did not follow the traditionally accepted definition of CCR as a “global” regulatory mechanism (84–86). These findings indicated the metabolic plasticity by which *G. daltonii* FRC-32 regulates its degradation pathways in response to dynamic environmental conditions (Fig. S4).

Our results suggested that benzoate transport and intracellular benzoate accumulation played a role in the regulatory mechanisms of genes involved in the benzoyl-CoA pathway during anaerobic benzoate oxidation in the presence of acetate. Inside the cell, benzoate likely facilitated metabolic readiness by inducing differential expression of genes *bamNOPQ* involved in the benzoyl-CoA pathway, thus resulting in differential benzoate oxidation in the presence of acetate. To the best of our knowledge, the present study is the first study to investigate benzoate transport in the family *Geobacteraceae* and to elucidate the essential role of benzoate transport in differential anaerobic benzoate oxidation in the presence of acetate.

## SUPPLEMENTAL MATERIAL

Supplemental materials spectrum.02324-24-s0001.pdf Tables S1 to S3, Figures S1 to S4, References, and Supplemental methods.

ASM does not own the copyrights to Supplemental Material that may be linked to, or accessed through, an article. The authors have granted ASM a non-exclusive, world-wide license to publish the Supplemental Material files. Please contact the corresponding author directly for reuse.
