# Supplementary material for: Differential anaerobic oxidation of benzoate in Geotalea daltonii FRC-32
Source: Microbiol Spectr. 2025 Mar 5;13(4):e02324-24. doi: 10.1128/spectrum.02324-24 (PMC11960108; doi:10.1128/spectrum.02324-24)
Supplement: Supplemental materials — Tables S1 to S3, Figures S1 to S4, References, and Supplemental methods. [file spectrum.02324-24-s0001.pdf]

Supplemental Materials for

Differential anaerobic oxidation of benzoate in *Geotalea daltonii*

Christina Kiessling, Sujay Greenlund, James Bullows, Cayden Samuels, Feranmi Aboderin, Nuria Ramirez, Kuk-Jeong Chin

This file includes:

1. Supplemental Materials and Methods
2. Tables S1-S3
3. Figures S1- S4
4. References used in Supplemental Materials

1. Supplemental Materials and Methods

Total cellular protein analysis by SDS-PAGE

G. daltonii FRC-32 cells were harvested, pelleted by centrifugation, resuspended in lysis buffer [50 mM Tris-HCl (pH 8.0), 20 mM NaCl, 5 µL/mg lysozyme, 5U DnaseI/ml (Roche)], boiled for 6 min and centrifuged for 1 min at 800 rpm. Subsequently, the supernatant was purified via TCA-acetone precipitation: supernatant was mixed with 10% TCA at a 1:1 ratio and incubated on ice for 30 min. Samples were centrifuged at 10.000 rpm at 4°C for 15 min. Supernatant was washed twice with ice-cold acetone and centrifuged at 10,000 rpm at 4 °C for 5 min. After the final centrifugation, pellets were resuspended in 1X NuPAGE sample buffer (Thermo Fisher Scientific, Wilmington, DE). For SDS-PAGE analysis of total cellular protein, protein samples were mixed with NuPAGE Reducing Agent (Thermo Fisher Scientific, Wilmington, DE). Samples were incubated at 95°C for 5 min and then placed on ice immediately. Hand-cast 5/10% Bis-Tris 10-well gels were used for SDS-PAGE analysis along with NuPAGE MOPS (Thermo Fisher Scientific, Wilmington, DE) running buffer with antioxidant added. The gel was stained with Coomassie Brilliant Blue (R-250) per standard procedure (BIO-RAD).

In silico protein structure prediction of putative benzoate transporter

In silico modeling of BenK's 3D protein structure was performed via AlphaFold network which is an Open-Source software that predicts the 3D coordinates of all heavy atoms for the protein of interest, using amino acid sequence as well as aligned sequences of homologues of the respective protein as input information (1). High accuracy of the structure predictions is attributed to innovative local refinement strategy that is achieved by breaking the structure, and feeding the refined structure components back into the network (1). To assess the accuracy and confidence for the structure predictions, AlphaFold provides a numerical confidence value which is depicted on a pLDDT scale ranging from 0 – 100 (1). The higher the numerical score, the higher is AlphaFold's confidence in the accuracy of the predicted structure (1).

In silico protein-ligand binding affinity prediction of putative benzoate transporter

To predict the protein-ligand binding affinity of the putative benzoate transporter BenK, AutoDock/Vina 1.1.2, an open-source program for performing molecular docking prediction, was used (2, 3). Both ligand and protein were uploaded in pdbqt format, a modified pdb format providing additional information necessary for binding predictions. A rectangular box was placed over the predicted binding site of the tested protein to precisely define the putative binding site. Upon binding site definition, docking runs were executed. Each ligand-protein binding prediction was executed three times. During the docking run, the interaction energy between each ligand atom and the receptor residue of the protein was calculated. Protein-ligand binding affinity predictions were represented as numerical values in kcal/mol; the lower the value, the higher the binding affinity (4). Additionally, Root mean square deviation (RMSD) values for each protein-ligand prediction were provided. To graph and statistically differentiate the predicted binding affinity of the different tested substrates to BenK, an RMSD value difference of 2 Angstrom compared to the

top prediction was used as a cutoff. Docking predictions with RMSD values of above 2 Angstrom are generally considered unsuccessful (5).

2. Supplemental Tables

Table S1. Primers used in this study

Primer*	Target gene	Sequence	Amplicon length (bps)
bamN-F	*bamN*	5′-CAAACCATCAACCGCCTCTG-3′	225
bamN-R		5′-ATAGGGTCGGTCATCTTGGGAT-3′	
bamO-F	*bamO*	5′-TCAAGGTCAACAGCAGCGG-3′	277
bamO-R		5′-TTGGCATAGGCGGCGATA-3′	
bamP-F	*bamP*	5′-CGGCACACTTCATCTGGC-3′	207
bamP-R		5′-CTGCCGTCATTGAGTCCGAT-3′	
bamQ-F	*bamQ*	5′-GCACCATCTGTCGTCAGCA-3′	226
bamQ-R		5′-CCGATGACGATGGCAAGA-3′	
recA-F	*recA*	5′-GTTGACCGCTATCATCAGTA-3′	275
recA-R		5′-AAATGCCCTCGCCGTAGAGAA-3′	
benK-F	*benK*	5′-TACTTCTTTGTGGTTATGGC-3′	280
benK-R		5′-CAGGTAAAGACGGTGGCTGTTA-3′	

*All primers listed were designed in this study.

Table S2. Genes encoding for proteins predicted to be involved in anaerobic degradation of aromatic compounds in *G. daltonii* FRC-32

**Gene name**	**Gene ID**	**Gene annotation**
bamO	Geob_0095	electron transfer flavoprotein β subunit
bamP	Geob_0096	electron transfer flavoprotein α subunit
bamQ	Geob_0097	6-hydroxycyclohex-1-ene-1-carbonyl-CoA dehydrogenase
bamR	Geob_0098	cyclohexa-1,5-dienecarbonyl-CoA hydratase
bamN	Geob_0100	thiolase
bamU	Geob_0101	metal-dependent hydrolase
xylR	Geob_0143	bacterial enhancer binding protein, sigma 54 dependent
bamV	Geob_0144	sensor histidine kinase
bamW	Geob_0145	sigma 54 dependent response regulator
benK	**Geob_0193**	**aromatic transporter**
bamY	Geob_0200	benzoyl-CoA ligase
bamA	Geob_0211	6-oxocyclohex-1-ene-1-carbonyl-CoA hydrolase
bamB1	Geob_0212	benzoyl-CoA reductase, bis-(molybdopterin)-oxotungsten-binding subunit
bamC1	Geob_0213	benzoyl-CoA reductase, iron-sulfur cluster-binding subunit
bamD1	Geob_0214	iron-sulfur cluster-binding oxidoreductase, CCG domain pair-containing, putative benzoyl-CoA reductase electron transfer
bamE1	Geob_0215	polyferredoxin, putative benzoyl-CoA reductase electron transfer protein
bamF1	Geob_0216	benzoyl-CoA reductase electron transfer protein, selenocysteine-containing, putative
bamG1	Geob_0217	benzoyl-CoA reductase electron transfer protein, putative, NADH-quinone oxidoreductase subunit E
bamH1	Geob_0218	benzoyl-CoA reductase electron transfer protein, putative, NADH-quinone oxidoreductase subunit F
bamI1	Geob_0219	iron-sulfur cluster-binding protein, putative
bamB3	Geob_0227	benzoyl-CoA reductase, bis-(molybdopterin)-oxotungsten-binding subunit
bamB4	Geob_0228	benzoyl-CoA reductase, bis-(molybdopterin)-oxotungsten-binding subunit
bamC2	Geob_0229	benzoyl-CoA reductase, iron-sulfur cluster-binding subunit
bamD2	Geob_0230	iron-sulfur cluster-binding oxidoreductase, CCG domain pair-containing, putative benzoyl-CoA reductase electron transfer protein
bamE2	Geob_0231	polyferredoxin, putative benzoyl-CoA reductase electron transfer protein
bamF2	Geob_0232	benzoyl-CoA reductase electron transfer protein, selenocysteine-containing, putative
bamG2	Geob_0233	benzoyl-CoA reductase electron transfer protein, putative, NADH-quinone oxidoreductase subunit E
bamH2	Geob_0234	benzoyl-CoA reductase electron transfer protein, putative, NADH-quinone oxidoreductase subunit F
bamI2	Geob_0235	iron-sulfur cluster-binding protein, putative

Table S3. Enzymes that are expressed during anaerobic benzoate oxidation in *G. daltonii* FRC-32

**Enzymes Unique to Benzoate Oxidation**	**Protein Size**
Histidine kinase BamV	67 kDa
Response regulator BamW	51 kDa
Bacterial enhancer binding protein XylR	66 kDa
Benzoate CoA-ligase BamY	58 kDa
**Benzoate transporter BenK**	**43 kDa**
Benzoate repressor BgeR	16 kDa

3. Supplemental Figures

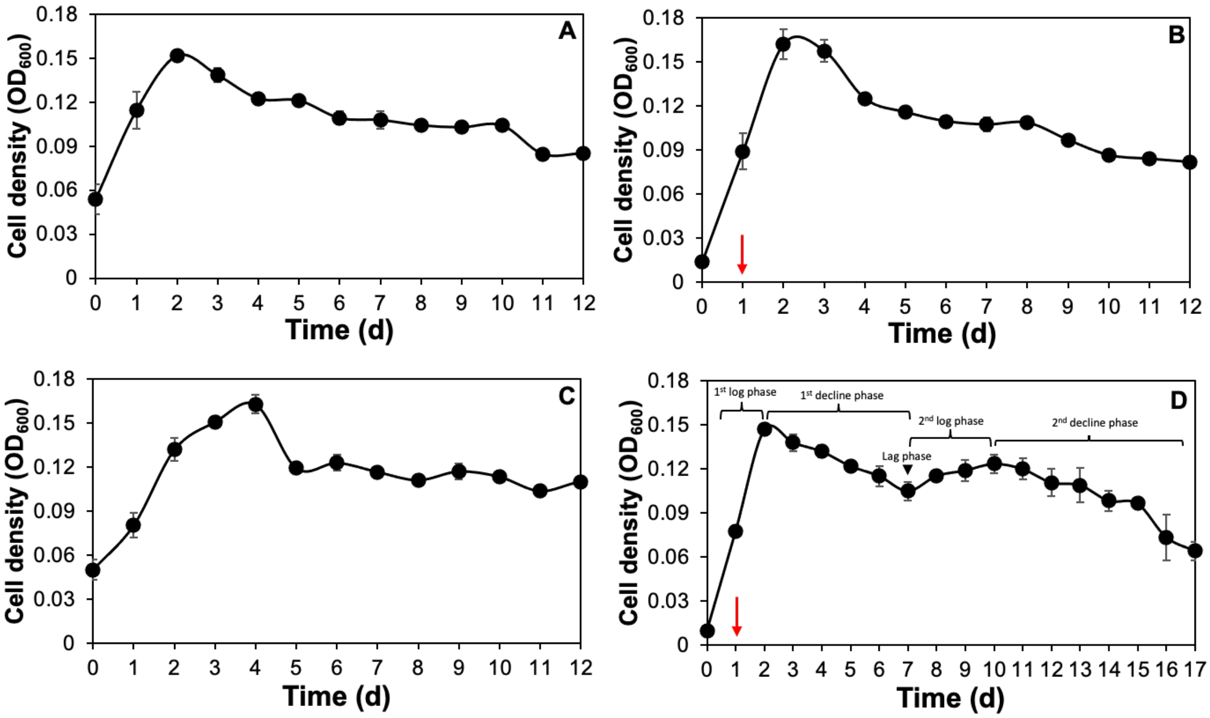

Figure S1. Growth characteristics of *G. daltonii* FRC-32 cultures grown on various carbon sources. A. Monoauxic growth in cultures grown on 1 mM benzoate + 2 mM acetate **B**. Monoauxic growth in cultures grown on 2 mM acetate spiked with 1 mM benzoate. **C.** Monoauxic growth in cultures grown on 1 mM benzoate + 5 mM acetate. **D.** Diauxic growth in cultures grown on 5 mM acetate spiked with 1 mM benzoate. Arrows indicate addition of benzoate after 1 day. The results represent the means ± standard errors of triplicate OD_{600} determinations of each sample obtained from triplicate cultures.

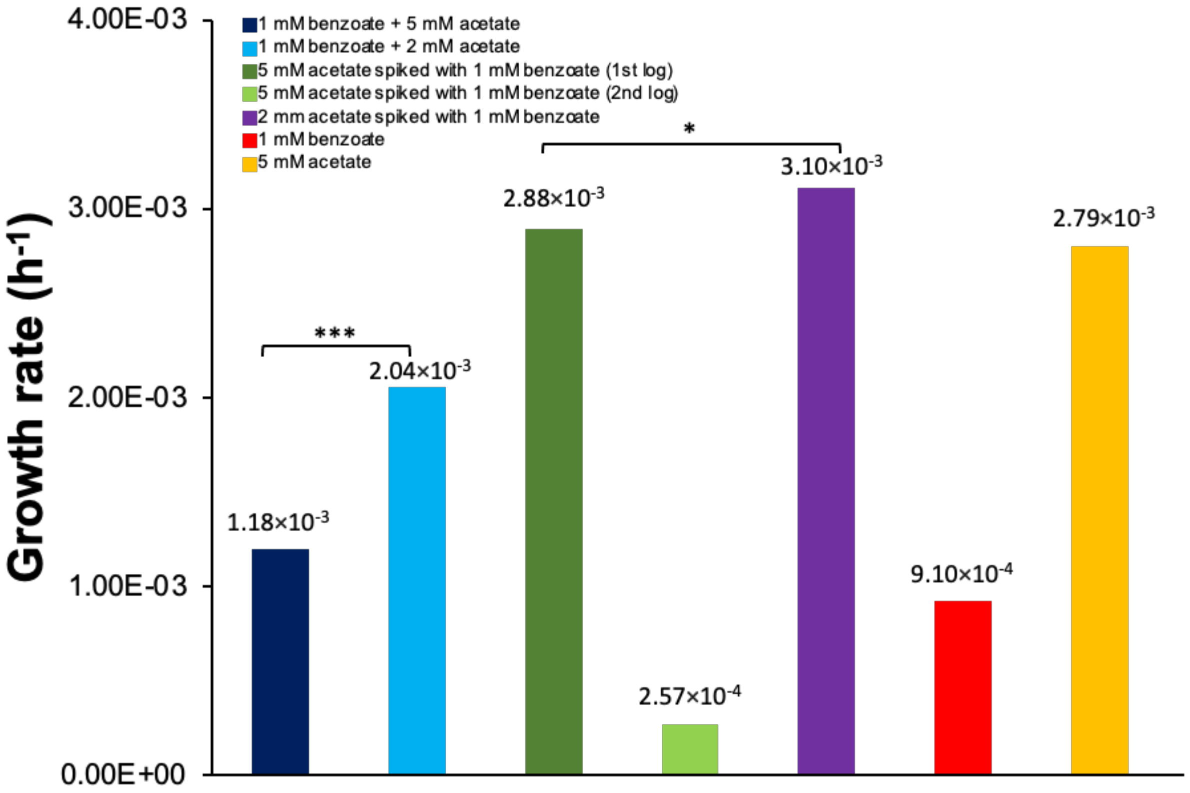

Figure S2. Growth rates in log phase of *G. daltonii* FRC-32 cultures grown on various carbon sources. Growth rates were calculated based on the change of cell density over time during the logarithmic phase of each growth curve displayed in Fig. 1A-D. The results represent the means ± standard errors of triplicate OD_{600} values from triplicate cultures (****P* >0.0005, **P*>0.05; as determined by Student's *t*-test). Significant differences compared to growth rate during growth on 1 mM + 5 mM acetate and 5 mM acetate spiked with 1 mM benzoate are indicated by asterisks (*** and *, respectively).

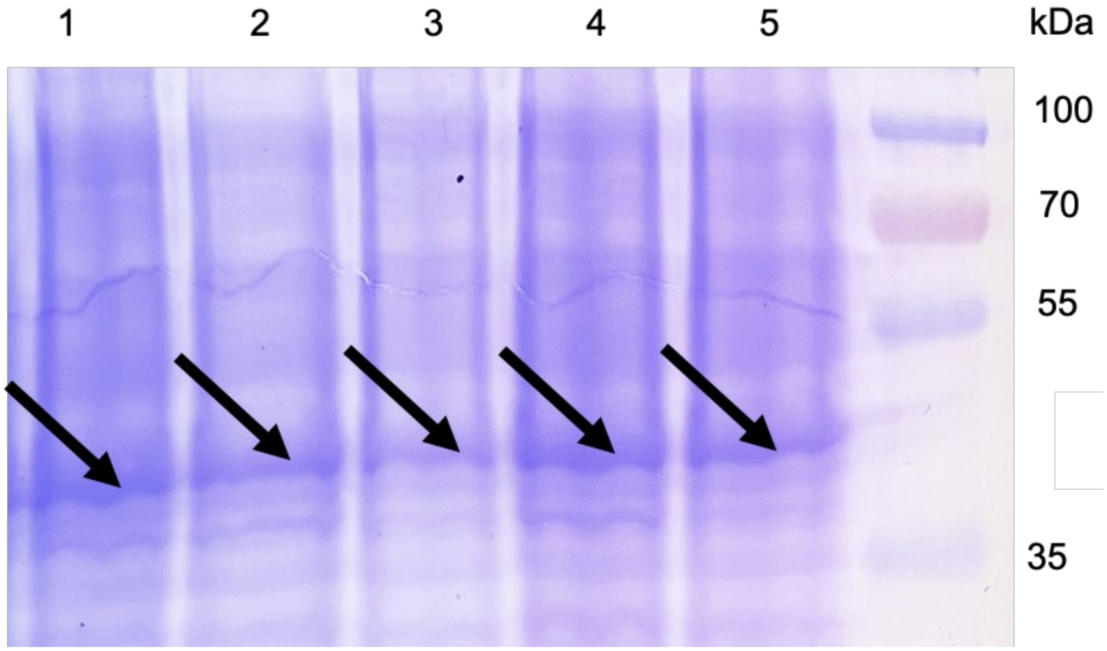

Figure S3. SDS-PAGE image showing the band indicative of putative benzoate transporter BenK in whole cell lysates from various growth phases *G. daltonii* FRC-32 cultures on acetate and benzoate. *Lane 1:* cells grown on 1 mM benzoate + 2 mM acetate; mid-log. *Lane 2:* cells grown on 2 mM acetate spiked with 1 mM benzoate, late-log. *Lane 3:* cells grown on 1 mM benzoate + 5 mM acetate; mid-log. *Lane 4:* cells grown on 5 mM acetate spiked with 1 mM benzoate, 1st late-log. *Lane 5:* cells grown on 5 mM acetate spiked with 1 mM benzoate, 2nd late log. Arrows indicate the location of the putative benzoate transporter BenK, *ca*. 43 kDa.

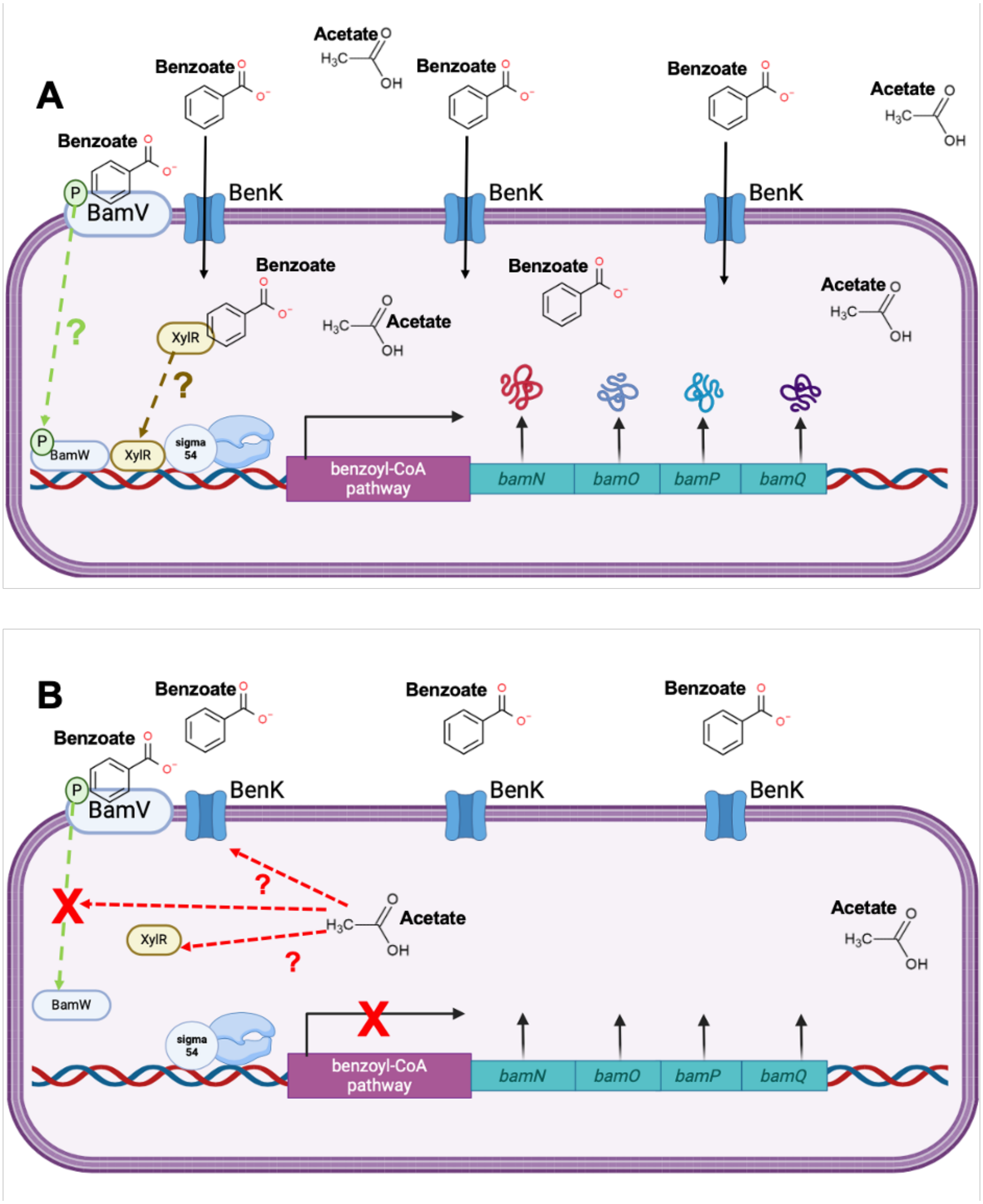

Figure S4. Proposed mechanism of differential anaerobic benzoate oxidation in the presence of acetate in *G. daltonii* FRC-32. A. Intracellular benzoate accumulation induced expression genes involved in benzoyl-CoA pathway, leading to simultaneous carbon source oxidation of benzoate and acetate. **B.** Sequential carbon source oxidation, possibly due to CCR.

4. References used in Supplemental Materials

1. Jumper J, Evans R, Pritzel A, Green T, Figurnov M, Ronneberger O, Tunyasuvunakool K, Bates R, Žídek A, Potapenko A, Bridgland A, Meyer C, Kohl SAA, Ballard AJ, Cowie A, Romera-Paredes B, Nikolov S, Jain R, Adler J, Back T, Petersen S, Reiman D, Clancy E, Zielinski M, Steinegger M, Pacholska M, Berghammer T, Bodenstein S, Silver D, Vinyals O, Senior AW, Kavukcuoglu K, Kohli P, Hassabis D. 2021. Highly accurate protein structure prediction with AlphaFold. Nature 596:583-589.
2. Trott O, Olson AJ. 2010. AutoDock Vina: Improving the speed and accuracy of docking with a new scoring function, efficient optimization, and multithreading. J Comput Chem 31:455-461.
3. Eberhardt J, Santos-Martins D, Tillack AF, Forli S. 2021. AutoDock Vina 1.2.0: New Docking Methods, Expanded Force Field, and Python Bindings. J Chem Inf Mod 61:3891-3898.
4. Seeliger D, de Groot BL. 2010. Ligand docking and binding site analysis with PyMOL and Autodock/Vina. J Comput Aided Mol Des 24:417-422.
5. Ding Y, Fang Y, Moreno J, Ramanujam J, Jarrell M, Brylinski M. 2016. Assessing the similarity of ligand binding conformations with the Contact Mode Score. Comput Biol Chem 64:403-413.
